# Loss of ACE2 Exacerbates Murine Renal Ischemia-Reperfusion Injury

**DOI:** 10.1371/journal.pone.0071433

**Published:** 2013-08-09

**Authors:** Fei Fang, George Chu Liu, Xiaohua Zhou, Stuart Yang, Heather Naomi Reich, Vanessa Williams, Amanda Hu, Janice Pan, Ana Konvalinka, Gavin Yadram Oudit, James William Scholey, Rohan John

**Affiliations:** 1 Departments of Medicine and Institute of Medical Science, University of Toronto, Toronto, Canada; 2 Division of Nephrology, Department of Medicine, University Health Network and University of Toronto, Toronto, Canada; 3 Division of Cardiology, Department of Medicine, Mazanlowski Alberta Heart Institute, University of Alberta, Edmonton, Canada; 4 Department of Pathology, University Health Network and University of Toronto, Toronto, Canada; University of Kentucky, United States of America

## Abstract

Ischemia-reperfusion (I/R) is a model of acute kidney injury (AKI) that is characterized by vasoconstriction, oxidative stress, apoptosis and inflammation. Previous studies have shown that activation of the renin-angiotensin system (RAS) may contribute to these processes. Angiotensin converting enzyme 2 (ACE2) metabolizes angiotensin II (Ang II) to angiotensin-(1–7), and recent studies support a beneficial role for ACE2 in models of chronic kidney disease. However, the role of ACE2 in models of AKI has not been fully elucidated. In order to test the hypothesis that ACE2 plays a protective role in AKI we assessed I/R injury in wild-type (WT) mice and ACE2 knock-out (ACE2 KO) mice. ACE2 KO and WT mice exhibited similar histologic injury scores and measures of kidney function at 48 hours after reperfusion. Loss of ACE2 was associated with increased neutrophil, macrophage, and T cell infiltration in the kidney. mRNA levels for pro-inflammatory cytokines, interleukin-1β, interleukin-6 and tumour necrosis factor-α, as well as chemokines macrophage inflammatory protein 2 and monocyte chemoattractant protein-1, were increased in ACE2 KO mice compared to WT mice. Changes in inflammatory cell infiltrates and cytokine expression were also associated with greater apoptosis and oxidative stress in ACE2 KO mice compared to WT mice. These data demonstrate a protective effect of ACE2 in I/R AKI.

## Introduction

Ischemia-reperfusion (I/R) is an important cause of acute kidney injury (AKI) and a common occurrence in volume depleted or septic patients and in the setting of organ procurement for transplant. I/R often leads to significant kidney damage including progressive chronic kidney disease (CKD) [1]–[3]. The pathophysiology of I/R injury is complex and includes the effects of hypoxia and cell death on various renal tubulointerstitial cells, vascular or hemodynamic factors, and inflammatory processes [4], [5]. Recent evidence suggests that the evolution and persistence of inflammation triggered by the initial non-immune insult plays a critical role in the outcome of I/R [6].

Activation of the renin-angiotensin system (RAS) is implicated in most forms of kidney injury, and inhibiting its main effector, angiotensin II (Ang II), remains a cornerstone of therapy for progressive CKD [7], [8]. The sequential action of renin and then angiotensin converting enzyme (ACE) on angiotensinogen and angiotensin I respectively produces Ang II, which contributes to vasoconstriction, local tissue oxidative stress, inflammation, and fibrosis in CKD [9], [10]. In a similar manner, the actions of Ang II may also contribute to I/R kidney injury. In previous studies of I/R injury in rats, the RAS was found to be activated and kidney Ang II levels increased after I/R [11]–[13].

The discoveries of ACE2, a homolog of ACE, and the Mas receptor, which binds angiotensin-(1–7) (Ang-(1–7)), have generated new interest in the RAS [14], [15]. ACE2 converts Ang II to Ang-(1–7), and the effects of Ang-(1–7) may oppose that of Ang II [16], [17]. In support of this concept, we and others have shown that genetic deletion or pharmacologic inhibition of ACE2 worsens experimental kidney disease including diabetic nephropathy and unilateral ureteral obstruction, while administration of recombinant ACE2 or over-expression of ACE2 improves kidney injury [18]–[23].

In the current study, we hypothesized that ACE2 would also be protective in kidney I/R. In order to test this hypothesis, we compared I/R-induced histopathologic injury and inflammation, apoptosis, and oxidative stress in wild-type mice and mice with a deletion in the *ace2* gene.

## Materials and Methods

### Ethics Statement

All experiments were conducted following the guidelines of the University of Toronto Animal Care Committee.

### Animals

Wild-type (WT) and *Ace2^−/y^* (ACE2 KO) mice on C57BL/6J background were generated as previously described [24], housed at the Division of Comparative Medicine at the University of Toronto, and fed standard mouse chow with free access to water. Only male mice were used in this study.

### Ischemia-Reperfusion

We first employed the unilateral model of I/R. 8-week old mice were administered analgesic (ketoprofen, 0.1 ml/10 g body weight) followed by anesthesia with inhaled isoflurane mixed with oxygen. Using a back incision, the left renal pedicle was exposed and the left renal artery constricted with 4–0 suture for 45 minutes and subsequently released. Vascular occlusion and release was confirmed by observing corresponding changes in kidney color. During the entire surgical procedure, animals were placed on a 37°C heating pad to maintain body temperature, and then allowed to recover under a warming light. Sham animals received an identical procedure without ligature. Mice were sacrificed 48 hours after surgery for tissue harvest, with body and kidney weights recorded. In order to obtain a better functional measure of injury, we studied the bilateral model of I/R. Using flank incisions, both renal pedicles were exposed and occluded with microaneurysm clamps (Roboz Surgical Instrument, Gaithersburg, MD, USA) for 25 minutes. Blood was collected at 48 hours after surgery for measurement of plasma BUN and creatinine.

### Blood Biochemistry

Blood samples were collected from carotid artery with Microvette® (Sarstedt Inc., Montreal, QC, CA) at time of sacrifice. Plasma was isolated by centrifuging blood samples at 2000 g for 5 min at room temperature, and stored at −80°C until use. Plasma blood urea nitrogen (BUN) and creatinine assessments were done at the Toronto Centre for Phenogenomics (Toronto, ON, CA).

### Histology and Immunohistochemistry

The left kidney (sham or I/R operated) was harvested and transversely sectioned into 3 approximately equal portions. The two polar portions were snap-frozen and the middle portion placed into 10% neutral buffered formalin (Sigma Aldrich, St Louis, MO, USA) for histology and immunohistochemistry analyses. Fixed kidney tissue was paraffin-embedded, sectioned, stained and scanned. 3-µm periodic acid-Schiff (PAS) stained sections were used to score histopathologic injury, which was done blinded to the experimental group. Tubular injury was assessed on a scale of 0 to 4 (0 being no injury and 4 being the most severely injured).

The following primary antibodies were used for immunohistochemistry: ACE (Santa Cruz Biotechnology, Santa Cruz, CA, USA), ACE2 (R&D, Minneapolis, MN, USA), neutrophil (AbD Serotec, Raleigh, NC, USA), F4/80 (AbD Serotec, Raleigh, NC, USA), CD3 (Dako Canada, Inc., Burlington, ON, CA), caspase-3 (Cell Signaling Technology, Inc., Danvers, MA, USA), Ki-67 (Dako Canada, Inc., Burlington, ON, CA) and nitrotyrosine (Millipore Biosciences Research Reagents (Chemicon), Temecula, CA, USA). TUNEL staining was performed according to a published protocol [25]. Endogenous peroxidase activity was inhibited with 3% hydrogen peroxide. Quantitation of neutrophils, CD3, TUNEL and Ki-67 positive cells was based on counting of positively staining nuclei by a Nuclear algorithm, and quantitation of macrophages and nitrotyrosine on positively stained area measured by a Positive Pixel Count algorithm of Aperio ImageScope software (Aperio Technologies, Inc., Vista, CA, USA). Caspase-3 positive cells were manually counted.

### Quantitative Real-time PCR

Snap-frozen mouse kidney tissue was homogenized in liquid nitrogen with pre-cooled pestle and mortar on dry ice, and total RNA extracted using RNeasy® Mini kit (Qiagen Inc., Mississauga, ON, Canada) following the manufacturer’s protocol. 1µg of the extracted RNA was reverse transcribed into cDNA with QuantiTech® Reverse Transcription Kit (Qiagen GmbH, Hilden, Germany) and used for quantitative real-time PCR. The TaqMan® Gene Expression Assay system (Applied Biosystems, Foster City, CA, USA) was used to perform real-time PCR with the following primers: *il1b* (Catalogue number (Cat#): Mm01336189_m1), *il6* (Cat#: Mm00446190_m1), *tnf* (Cat#: Mm99999068_m1), *cxcl2* (MIP-2, Cat#: Mm00436450_m1), *ccl2* (MCP-1, Cat#: Mm00441242_m1), *bax* (Cat#: Mm00432051_m1), *bcl2* (Cat#: Mm00477631_m1), *ace* (Cat#: Mm00802048_m1), *ace2* (Cat#: Mm01159006_m1), *agt* (angiotensinogen, Cat#: Mm00599662_m1), *ren1* (renin, Cat#: Mm02342884_g1), *agtr1a* (angiotensin II receptor type 1 Cat#: Mm00616371_m1), *mas1* (Cat#: Mm00434823_s1). 18s (Cat#: Hs99999901_s1) was used as internal control.

### Western Blot

The following antibodies were used: anti-Bcl-2 antibody (#3498) (Cell Signaling Technology, Inc., Danvers, MA, USA); anti-Bax antibody (ab10813) (Abcam Inc., Cambridge, MA, USA); β-Actin (Santa Cruz Biotechnology, Inc. Santa Cruz, CA, USA). Snap-frozen kidney tissue was homogenized by sonification in lysis buffer (Cell Signaling Technology, Inc., Danvers, MA, USA). Proteins in tissue lysates were separated by 10% SDS-PAGE gel, blotted onto PVDF membrane and detected with an enhanced chemiluminescence system (ECL) kit (Millipore Corp., Billerica, MA, USA). Densitometry measurement was calculated by Scion Image software (Scion Corp. Frederick, MD, USA).

### Angiotensin II Peptide Measurement

The concentration of renal parenchymal Ang II was determined by an Angiotensin II EIA kit (Peninsula Laboratories, LLC, San Carlos, CA, USA). According to the manufacturer, the Ang II-binding antibody does not cross-react with Ang 1 or Ang (1–7). Tissue was prepared as follows: snap-frozen mouse kidney tissue was homogenized in ice-cold methanol on ice and centrifuged at 12000 g, 4°C for 10 minutes. The supernatant was collected and dried by centrifugal evaporation. Dried samples were reconstituted with the EIA buffer supplied by the manufacturer and used for Ang II measurement. Protein concentrations were determined by the Bradford assay (Bio-Rad Laboratories, Inc., Hercules, CA, USA) from the reconstituted samples and used for normalization.

### Statistical Analysis

Unless specified otherwise, results are expressed as mean ± SE. One-way ANOVA with Bonferroni post-hoc test was used for comparison of multiple groups. All statistical analyses were done with GraphPad Prism software (GraphPad Software, Inc., La Jolla, CA, USA), and statistical significance defined as p<0.05.

## Results

### Whole Animal, Macroscopic and Microscopic Kidney Examination

Compared to corresponding sham animals, there was no significant change in body weight after I/R in either WT mice or ACE2 KO mice (**Table 1**). Kidney weight to body weight ratio increased in both groups after I/R (**Table 1**). BUN and creatinine levels were measured to assess kidney function. Plasma BUN was modestly elevated to a similar extent in both WT and ACE2 KO animals after I/R, while plasma creatinine values were unchanged (**Table 1**).

**Table 1:** Whole Animal Data.

Parameter	WT sham	ACE2 sham	WT IR	ACE2 IR
BW (g)	23.71±0.55	21.83±1.27	22.98±0.45	21.06±0.29*
LKW (g)	0.1422±0.0040	0.1350±0.0110	0.1741±0.0076*	0.1723±0.0066
LKW/BW	0.0060±0.0001	0.0061±0.0001	0.0076±0.0004*	0.0082±0.0004*
BUN (mmol/L)	8.4±0.52	9.3±0.64	11.4±0.47*	12.6±0.80*
Creatinine(µmol/L)	21±0.8	21±1.4	22±1.1	22±1.4

BW (Body weight) and LKW (left kidney weight) were recorded at time of sacrifice 48 hours after surgery for all experimental groups. BUN and creatinine levels were measured with frozen plasma samples. Results are shown as mean ± SE. For BW, LKW AND LKW/BW, n = 8 for WT sham; n = 8 for ACE2 sham; n = 12 for WT IR; n = 11 for ACE2 IR. For BUN and creatinine, n = 6 for all groups.

* p<0.05 compared to WT sham.

**Figure 1:**
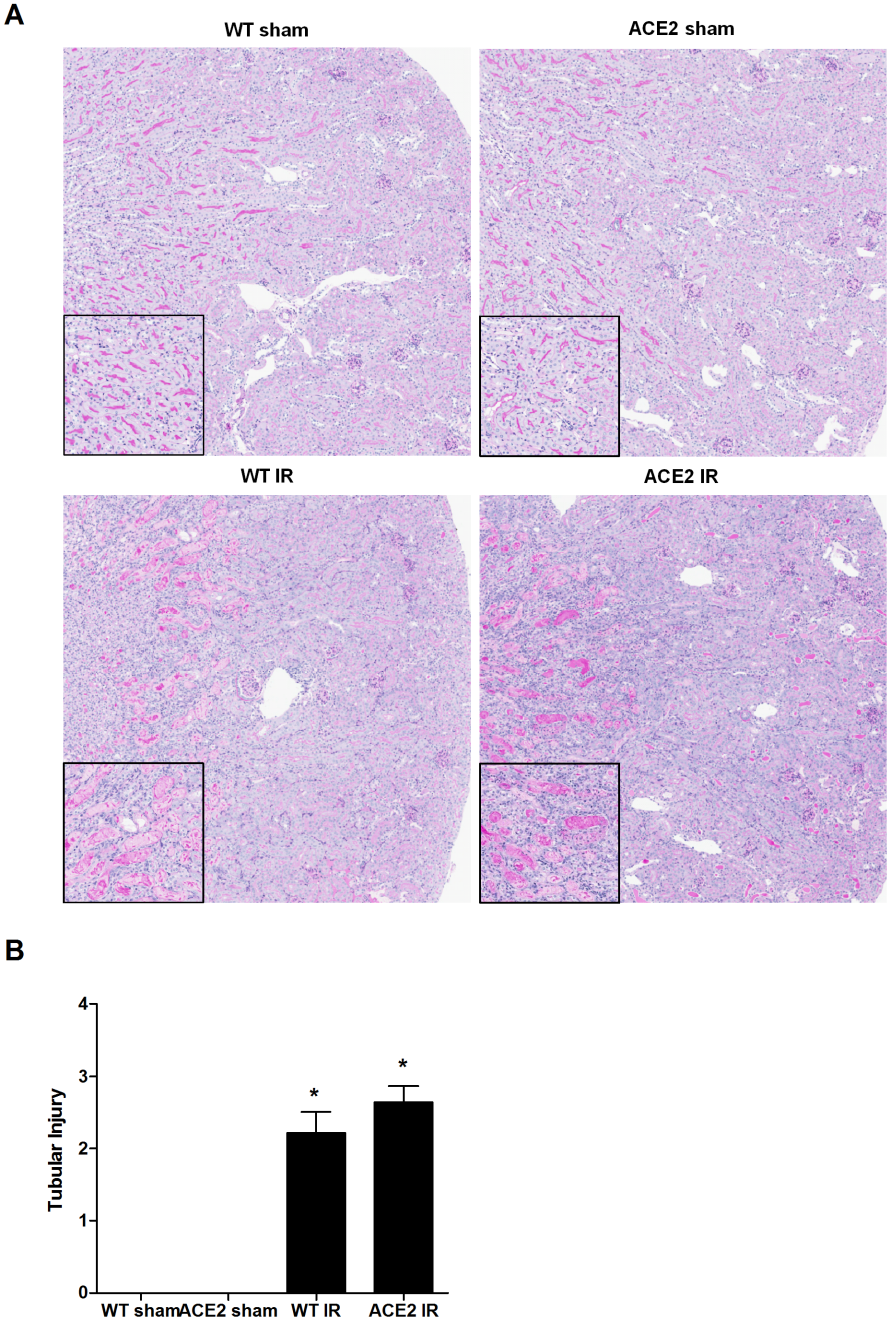
Histopathologic changes after renal I/R. (**A**) Representative images of PAS stained kidney sections from wild-type (WT) and ACE2 knock-out mice (ACE2 KO) after sham or I/R surgery, magnification: 50x; insert: high power field of the cortico-medullary junction, magnification: 200×. (**B**) Tubular injury score based on PAS sections for each experimental group, on a scale of 0 to 4. Results are presented as mean ± SE. n = 8 for WT sham and ACE2 sham; n = 12 for WT IR; n = 11 for ACE2 IR. *p<0.0001 vs. WT sham.

Histopathologic injury after I/R was assessed in PAS-stained sections. Injury was confined to the tubulointerstitial compartment and most pronounced in the outer medullary region, and included areas of tubular necrosis (**Figure 1A**). The inner medulla was the area next most affected, albeit to a much lesser extent, followed by the renal cortex. The mean value for tubular injury score after I/R tended to be higher in the ACE2 KO mice compared to the WT mice, but the difference did not reach statistical significance (**Figure 1B**). There was also no difference between the groups when injury scores were limited to the outer medulla (data not shown).

### Inflammatory Cell Infiltration and Pro-inflammatory Cytokine Expression

**Figure 2:**
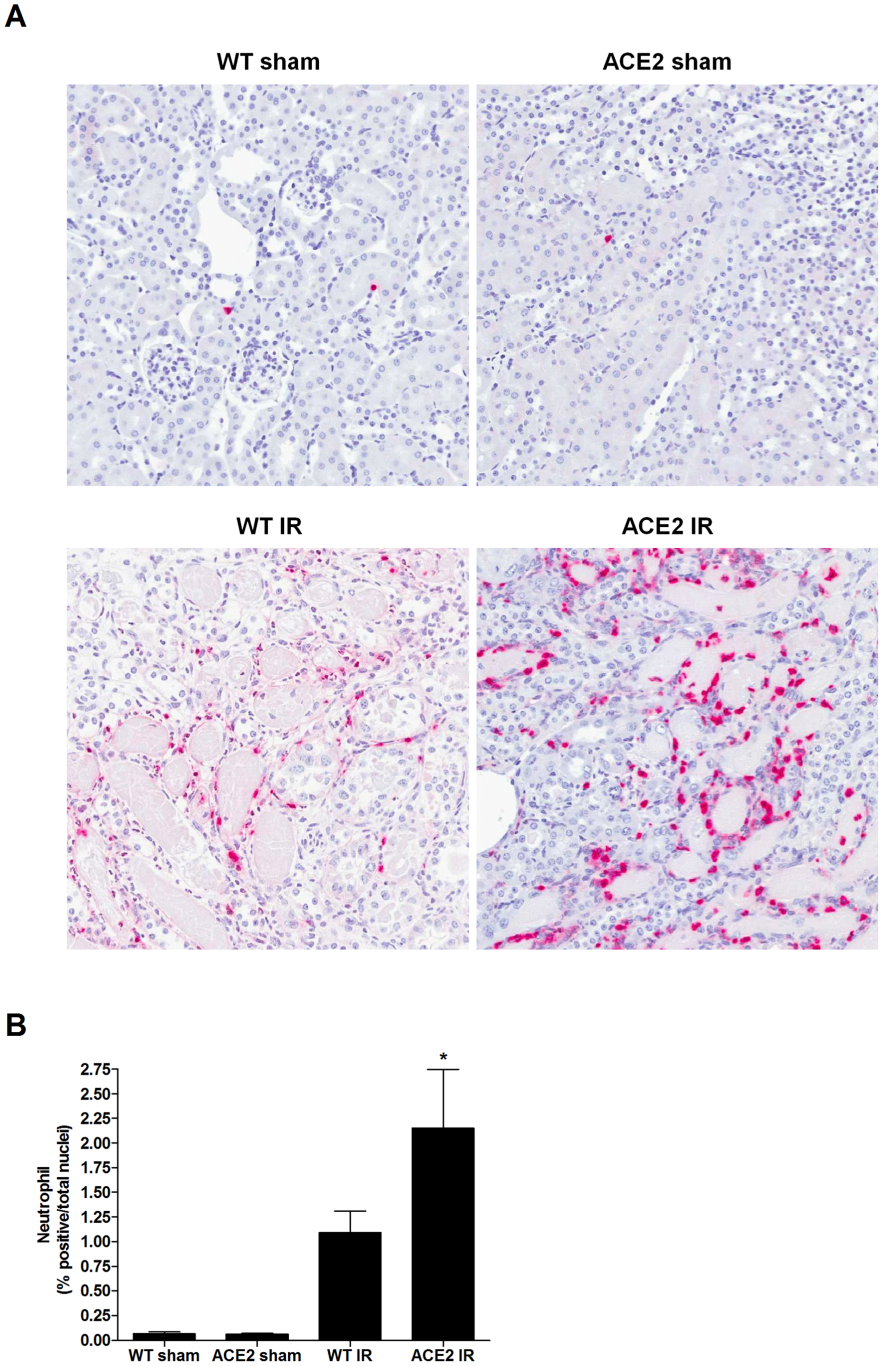
Kidney neutrophil infiltration following I/R. (**A**) Representative images of anti-neutrophil staining of kidney sections from WT sham, ACE2 sham, WT IR and ACE2 IR mice; magnification: 200x. (**B**) Quantitation of neutrophil infiltration using ImageScope Nuclear algorithm. Results are presented as mean ± SE. n = 5 for WT sham; n = 8 for ACE2 sham; n = 8 for WT IR; n = 9 for ACE2 IR. * p<0.05 vs. WT sham.

**Figure 3:**
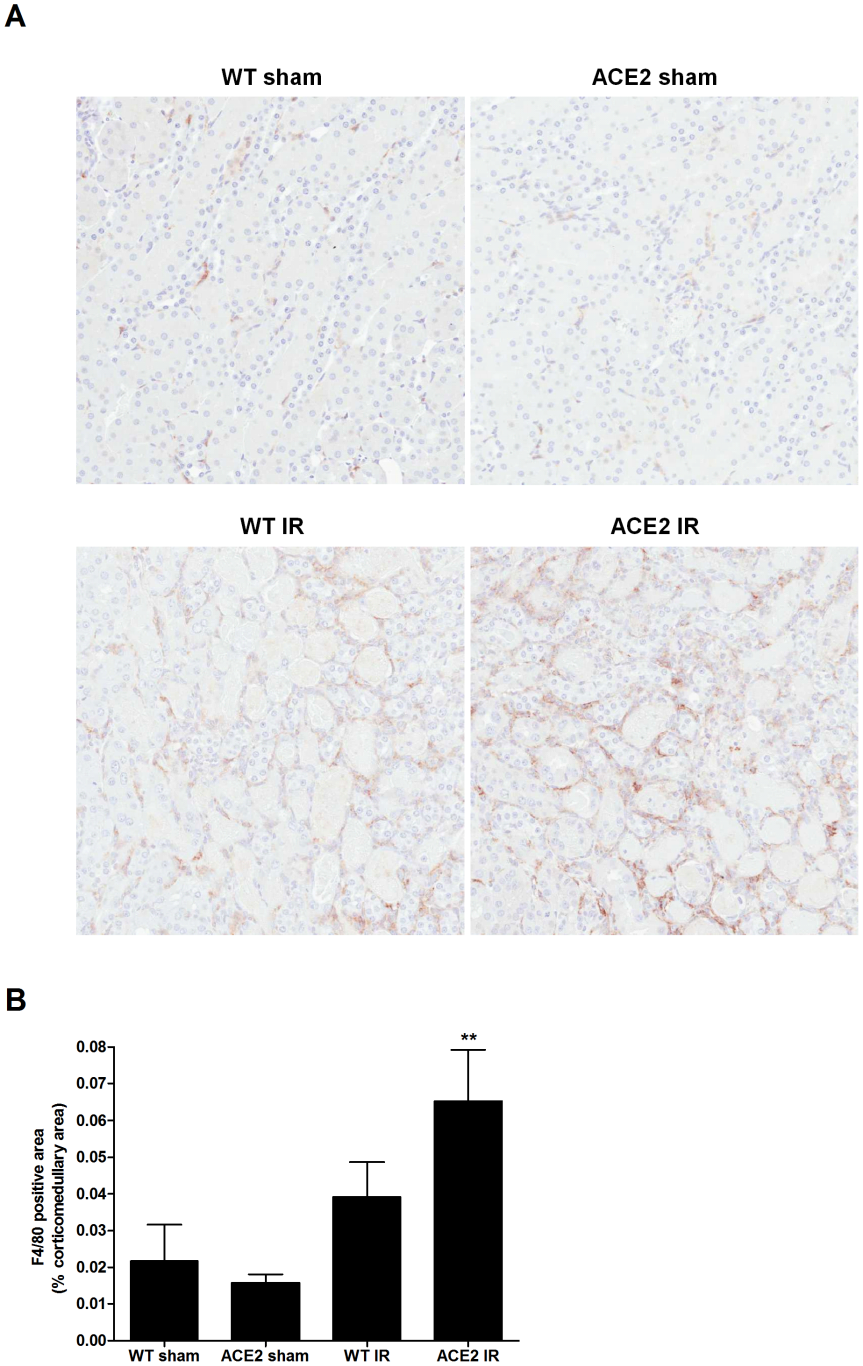
Macrophage (F4/80 positive cell) infiltration after I/R. (**A**) Representative images of F4/80 staining of kidney sections from WT and ACE KO mice after sham or I/R surgery; magnification: 200x. (**B**) Percentage of F4/80 positive area in cortico-medullary region calculated by ImageScope Positive Pixel Count algorithm. Results are presented as mean ± SE. n = 4 for WT sham; n = 7 for ACE2 sham; n = 8 for WT IR; n = 9 for ACE2 IR. **p<0.05 vs. ACE2 sham.

**Figure 4:**
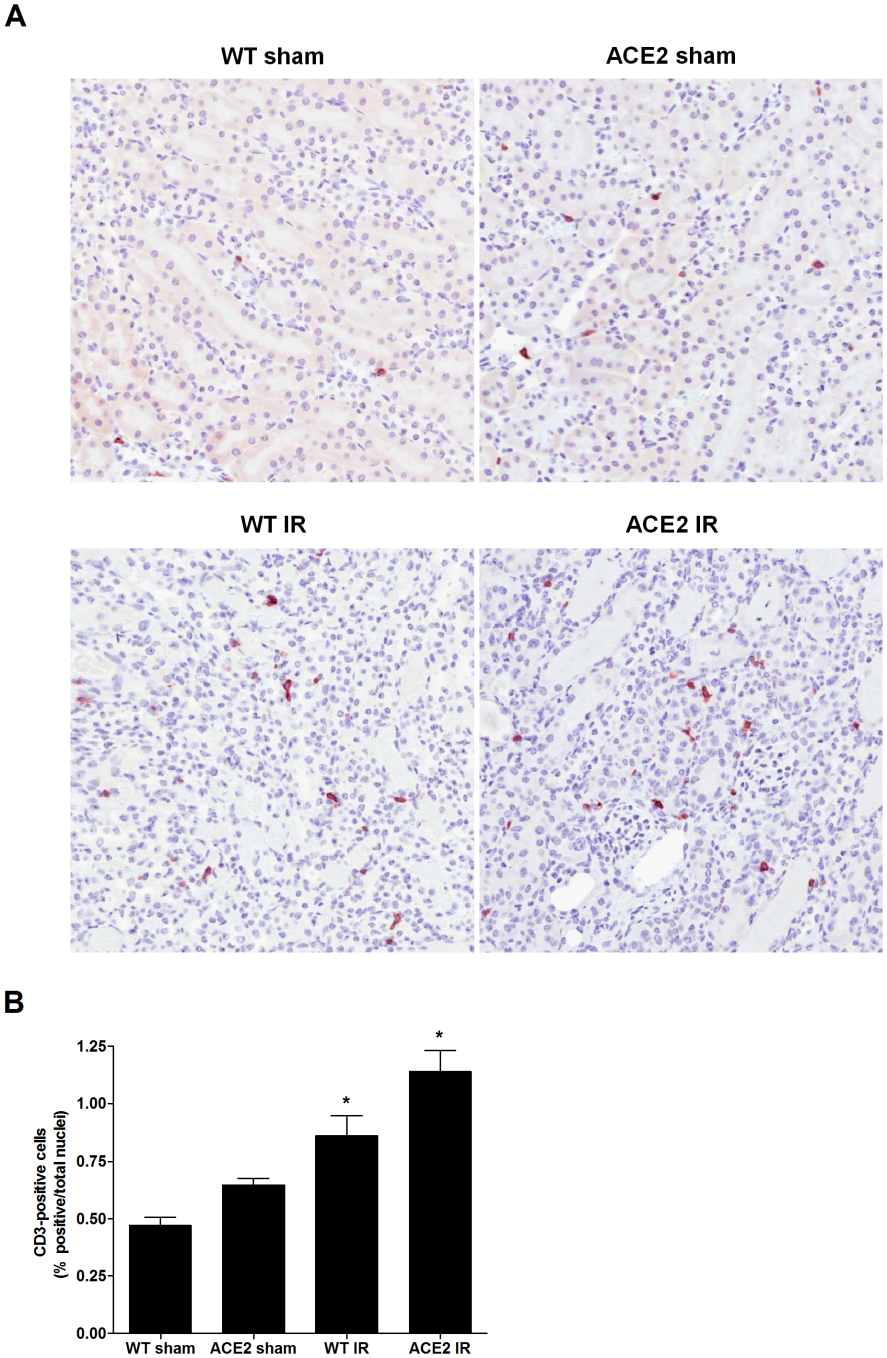
T cell (CD3 positive cell) infiltration following I/R. (**A**) Representative images of CD3 staining of kidney sections from WT sham, ACE2 sham, WT IR and ACE2 IR mice; magnification: 200x. (**B**) Quantitation of T cell infiltration using ImageScope Nuclear algorithm. Results are presented as mean ± SE. n = 8 for WT sham and ACE2 sham; n = 9 for WT IR; n = 11 for ACE2 IR. * p<0.05 vs. WT sham.

Neutrophils, F4/80 positive cells (macrophages) and CD3 positive cells (T cells) were assessed as indicators of renal parenchymal immune cell infiltration. **Figures 2** **,** **3** **and** **4** show the marked neutrophil, macrophage, and T cell infiltration within the kidneys of mice after I/R. Mean values for neutrophil infiltration were significantly higher in the ACE2 KO mice subjected to I/R compared to WT mice (p<0.05) (**Figure 2B**). A similar trend was seen for the numbers of macrophages (**Figure 3B**) and CD3-positive cells (**Figure 4B**), although the differences did not reach statistical significance.

**Figure 5:**
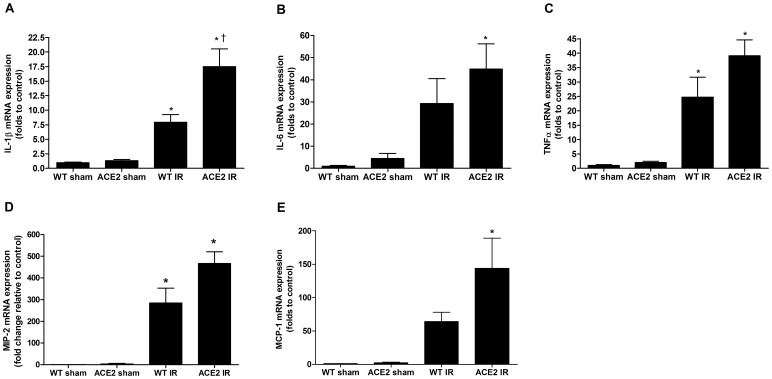
Pro-inflammatory cytokine and chemokine levels after I/R. mRNA expression levels of IL-1β (**A**), IL-6 (**B**), TNFα (**C**), MIP-2 (**D**) and MCP-1 (**E**), in WT and ACE2 KO mouse kidney after sham or I/R surgery. 18s was used as internal control. Results are normalized to WT sham and presented as mean ± SE. n = 5 for WT sham; n = 6 for ACE2 sham; n = 6 for WT IR; n = 5 for ACE2 IR. * p<0.05 vs. WT sham. ^†^ p<0.05 vs. WT IR.

Measurement of mRNA levels of pro-inflammatory cytokines, IL-1β, IL-6, and TNFα, and chemokines, MIP-2 and MCP-1 showed dramatic induction of all genes after I/R (**Figure 5**). Additionally, mean mRNA levels of all cytokines after I/R were higher in the kidneys of the ACE2 KO mice compared to the kidneys of WT mice with a statistically significant difference for IL-1β and MCP-1 (**Figure 5** **D, E**).

### Detection of Apoptosis, Cell Proliferation and Oxidative Stress

**Figure 6:**
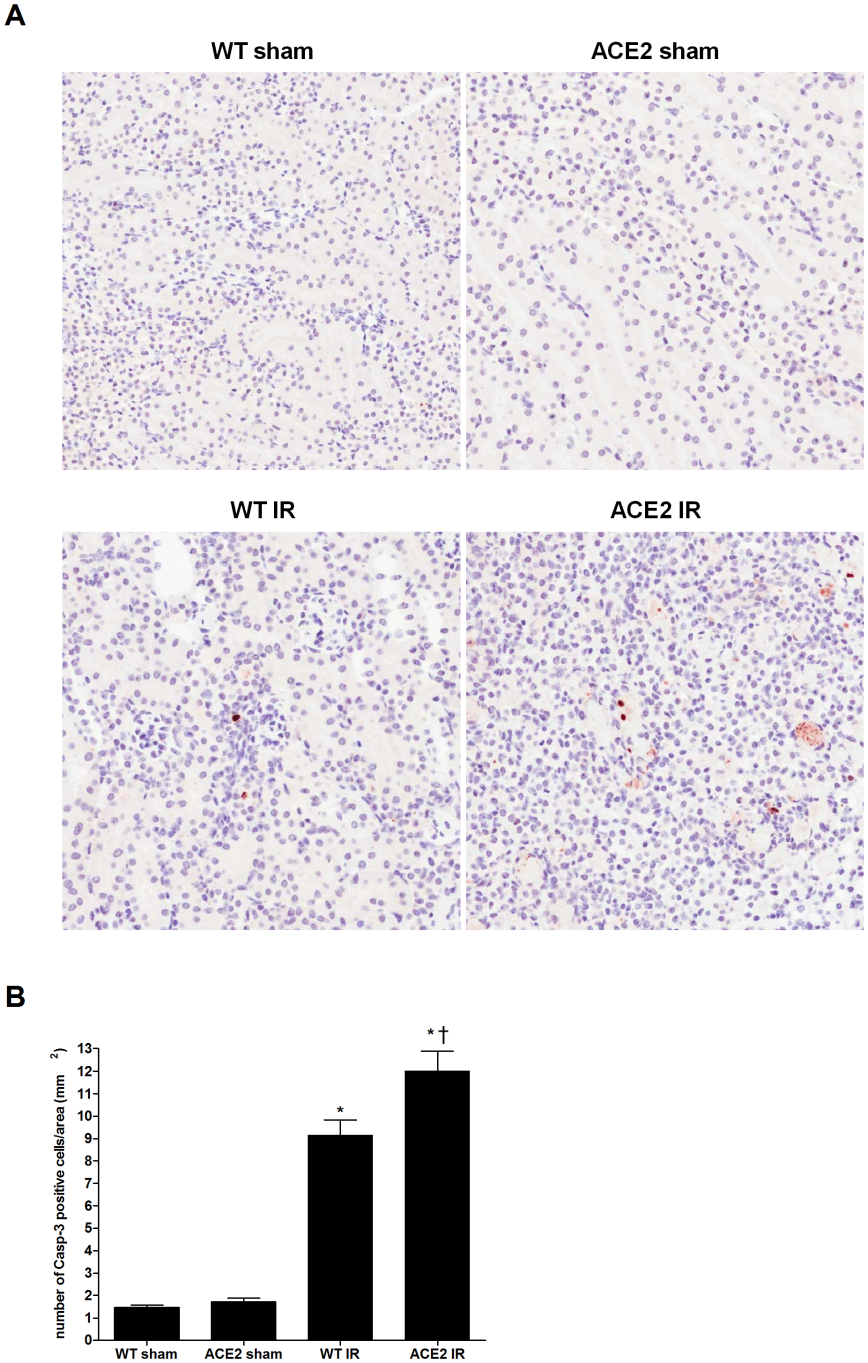
Immunohistochemistry for cleaved caspase-3 following I/R. (**A**) Representative images of caspase-3 staining in kidney sections from WT sham, ACE2 sham, WT IR and ACE2 IR mice; magnification: 200x. Positively staining cells were counted in randomly chosen areas, and the number of positive cells and size of each area were recorded. (**B**) Quantitation of caspase-3 staining by numbers of positive cells per mm^2^ of tissue. Results are presented as mean ± SE. n = 8 for WT sham; n = 7 for ACE2 sham; n = 12 for WT IR; n = 10 for ACE2 IR. * p<0.05 vs. WT sham. ^†^ p<0.05 vs. WT IR.

**Figure 7:**
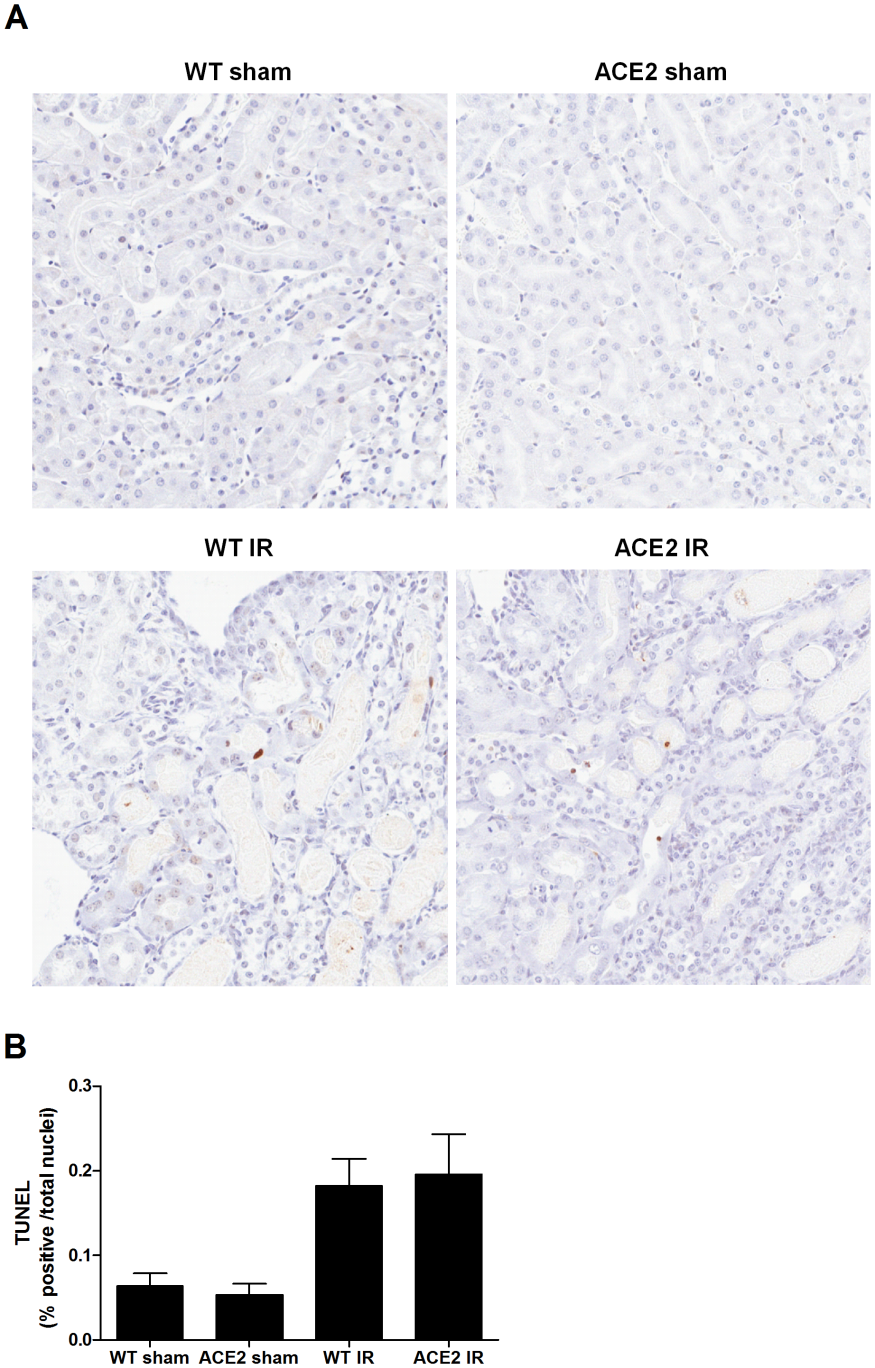
Immunohistochemical TUNEL staining after I/R. (**A**) Representative images of TUNEL staining on kidney sections in WT and ACE2 group after sham or I/R operation; magnification: 200x. (**B**) Quantification of positively stained cells as determined by ImageScope Nuclear algorithm. Results are presented as mean ± SE. n = 5 for WT sham; n = 6 for ACE2 sham; n = 7 for WT IR; n = 9 for ACE2 IR.

There was an almost five-fold increase in the number of apoptotic cells (caspase 3-positive cells) in the WT mice after I/R compared to the sham-operated mice (**Figure 6** **)**. Furthermore, the mean number of caspase 3-positive cells was significantly higher in ACE2 KO mice after I/R compared to WT mice. Compared to caspase-3, TUNEL staining showed greater heterogeneity within groups in the numbers of positive cells. Mean values of TUNEL-positive cells appeared similarly increased after I/R in the WT and ACE2 KO mice (**Figure 7**). This may be due to a decrease in specificity of the TUNEL stain when there is both apoptosis and necrosis as occurs in I/R injury [26].

**Figure 8:**
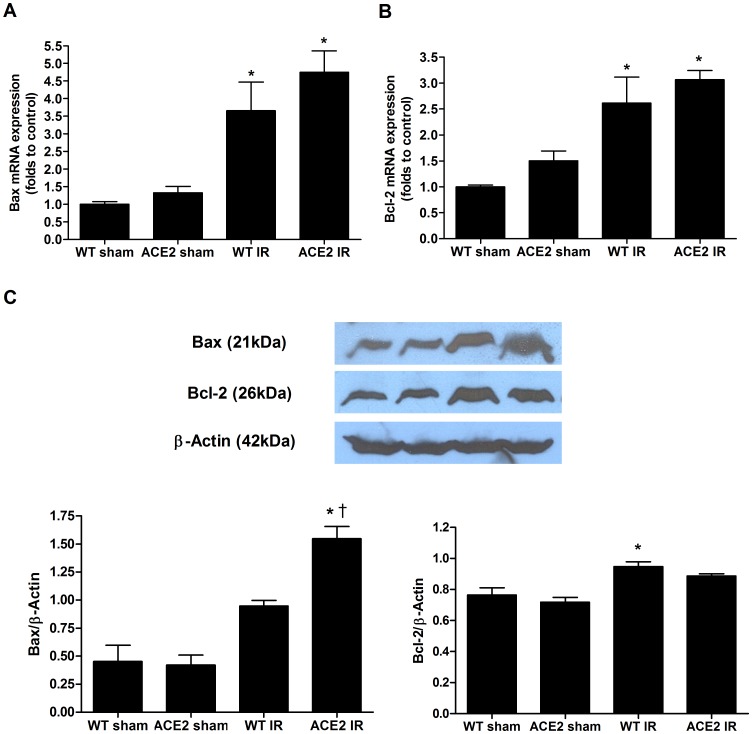
Bcl-2 and Bax expression following I/R. mRNA levels of Bax (**A**) and Bcl-2 (**B**) were determined by real-time PCR. 18s was used as internal control. Results were normalized to WT sham. (**C**) Protein expressions of Bax and Bcl-2 were measured by western blot. Representative images and densitometries are provided. β-Actin was used as loading control. Results are presented as mean ± SE. For real-time PCR, n = 5 for WT sham; n = 5 for ACE2 sham; n = 6 for WT IR; n = 5 for ACE2 IR. For western blot, experiments were performed in triplicates. * p<0.05 vs. WT sham. ^†^ p<0.05 vs. WT IR.

mRNA levels of Bax and Bcl-2 were increased after I/R (**Figure 8** **A, B**). A pro-apoptotic phenotype was more evident in the ACE-2 KO group at the protein level with a significant increase in Bax in the ACE-2 KO mice after I/R and no difference between the groups in Bcl-2 expression (**Figure 8C**).

**Figure 9:**
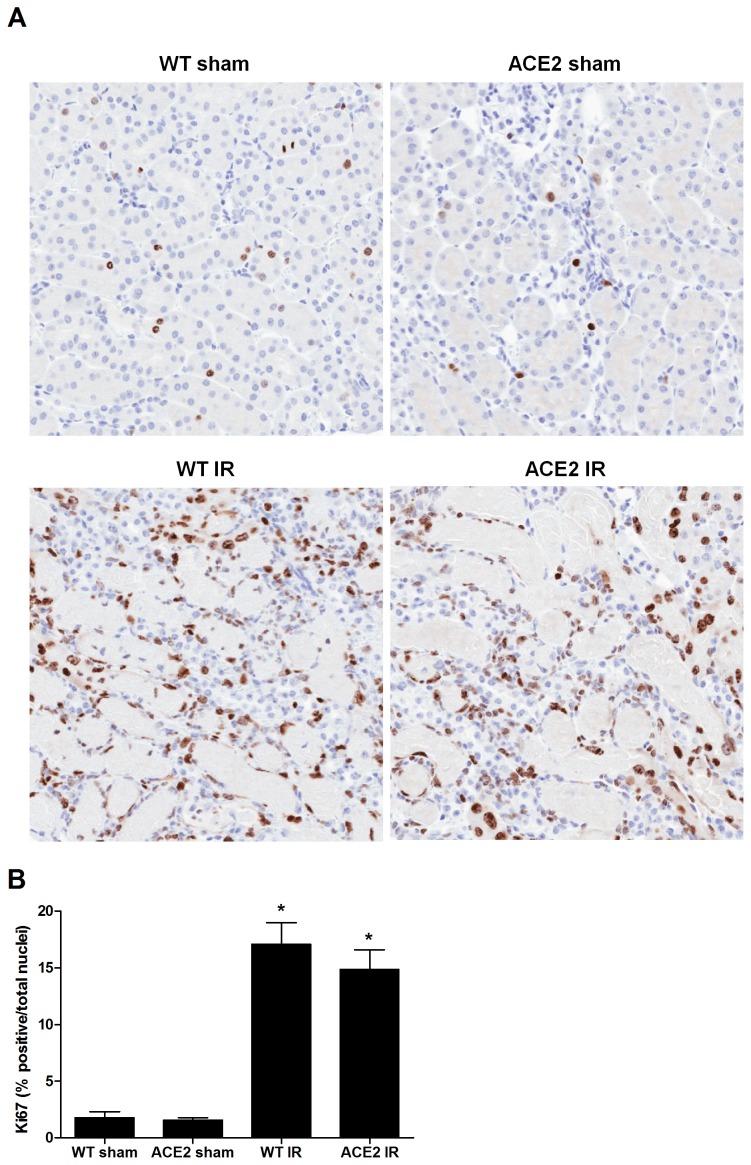
Cellular proliferation after I/R. (**A**) Representative images of Ki-67 staining on kidney sections from WT sham, ACE2 sham, WT IR and ACE2 IR mice. Magnification: 200x. (**B**) Percentage of positively stained cells (positive cells/total cells) as determined by ImageScope Nuclear algorithm. Results are shown as mean ± SE. n = 5 for WT sham; n = 7 for ACE2 sham; n = 8 for WT IR; n = 9 for ACE2 IR. * p<0.05 vs. WT sham.

As a measure of recovery from I/R injury, we assessed cellular proliferation by immunohistochemistry for Ki-67. As expected, the numbers of proliferating cells were markedly increased after I/R (**Figure 9**). Numbers of Ki-67 cells were slightly lower in the ACE-2 KO compared to WT mice, but mean values were not statistically different between the groups (**Figure 9B**).

**Figure 10:**
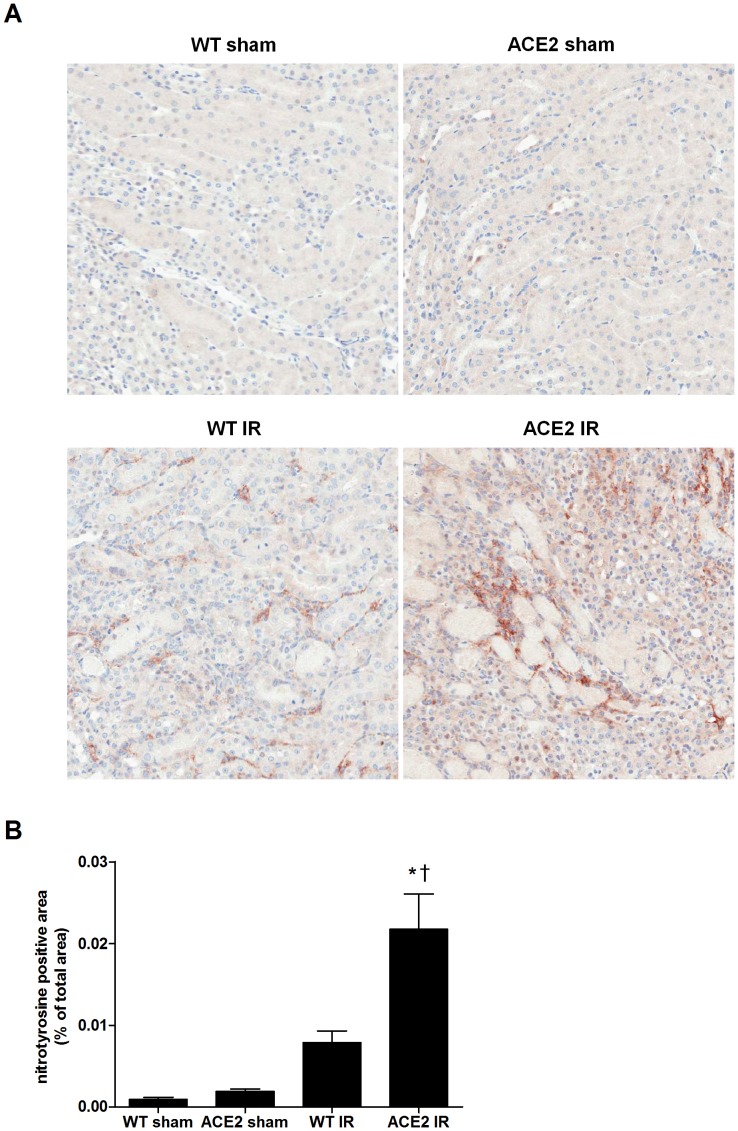
Induction of oxidative stress following I/R. (**A**) Representative images of nitrotyrosine immunostaining in kidney sections from WT and ACE2 KO mice after sham or I/R surgery; magnification: 200x. (**B**) Quantitation of nitrotyrosine staining by positive area per total area using Image Scope Positive Pixel Count algorithm. Results are presented as mean ± SE. n = 5 for WT sham; n = 7 for ACE2 sham; n = 8 for WT IR; n = 11 for ACE2 IR. * p<0.05 vs. WT sham. ^†^ p<0.05 vs. WT IR.

In order to assess oxidative stress, we measured nitrotyrosine staining in kidney tissue. There was an increase in nitrotyrosine staining after I/R in both groups of mice with significantly greater staining in ACE2 KO mice compared to WT mice (**Figure 10**).

### Expression of RAS Components and Renal AngII Level

**Figure 11:**
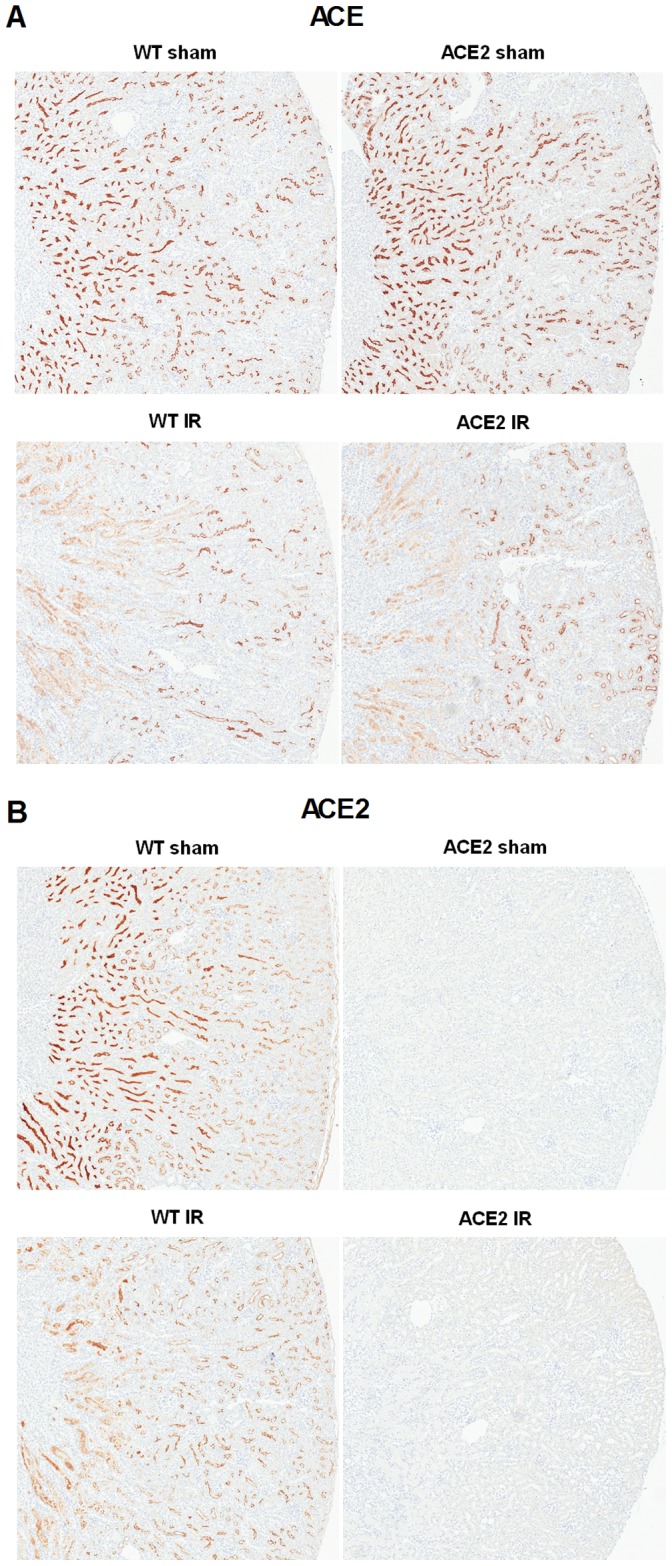
Immunohistochemical staining for ACE and ACE2. Representative images of ACE (**A**) and ACE2 (**B**) stained kidney sections in WT and ACE2 KO mice after sham or I/R surgery. Magnification: 50X.

In order to relate I/R-induced kidney injury to activation of the RAS, we measured the intra-renal expression of RAS components. In sham mice, immunohistochemistry for ACE showed strong staining along the apical border of the proximal tubules (**Figure 11A**). Staining was strongest in the cortico-medullary junction area and slightly weaker and patchy in the outer cortex. Following I/R, staining for ACE was decreased in both WT and ACE2 KO mice. The decrease was most pronounced in the corticomedullary area corresponding with severe tubular injury. Immunohistochemistry for ACE2 showed similar staining as for ACE in the WT sham mice (**Figure 11B**). After I/R, WT mice showed decreased ACE2 staining in the corticomedullary area but slightly increased staining in the outer cortex.

**Figure 12:**
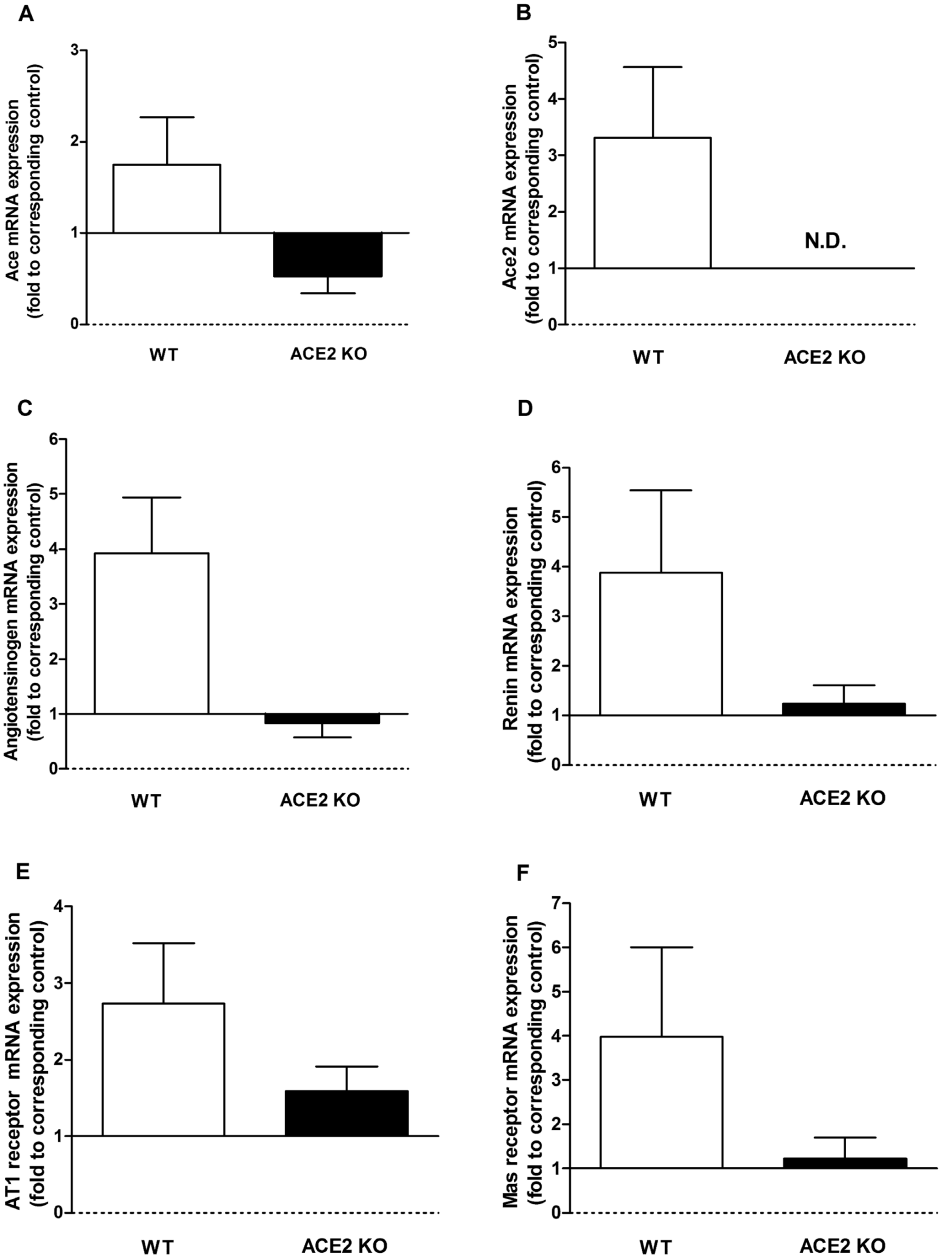
Changes in expression levels of RAS components after renal I/R. Expression of *ace* (**A**), *ace2* (**B**), *angiotensinogen* (**C**), *renin* (**D**), *agtr1* (angtiotensin II receptor, type I,) (**E),** and *mas* (**F**) after I/R were compared to corresponding control sham animals in WT and ACE2 KO groups. mRNA levels were determined by real-time PCR and 18s used as internal control. Results are presented as mean ± SE. n = 6 in WT group; n = 4 in ACE2 group.

As expected, ACE2 was not detected in the two groups of ACE2 KO mice by immunohistochemistry or mRNA measurements. There was no significant difference in the expression of any of the other RAS components between the WT and ACE2 KO sham groups (**Table 2**). As illustrated in **Figure 12**, WT mice showed a trend towards increased expression of all RAS components after I/R. In the ACE2 KO mice, I/R produced little or no change in mRNA levels of angiotensinogen, renin and Mas receptor, a small decrease in ACE and a small increase in AT1 receptor.

**Table 2:** mRNA levels of components of the renin-angiotensin system in WT and ACE2 KO mice after sham operation or I/R.

Components	WT sham (n = 5)	ACE2 sham (n = 5)	WT IR (n = 7)	ACE2 IR (n = 6)
ACE	0.70±0.06	0.89±0.09	1.06±0.35	0.36±0.12
ACE2	0.74±0.04	N.D.	2.13±0.85	N.D.
Angiotensinogen	0.69±0.06	1.07±0.23	2.37±0.69	0.85±0.18
Renin	0.68±0.08	1.01±0.19	2.31±1.00	1.62±0.32
AT_1_R	0.73±0.06	0.67±0.07	2.31±0.71	1.21±0.17
Mas	0.68±0.12	0.84±0.10	1.14±0.33	0.78±0.30

Values are expressed as fold change versus WT sham and presented as mean ± SE. N.D., non-detectable.

**Figure 13:**
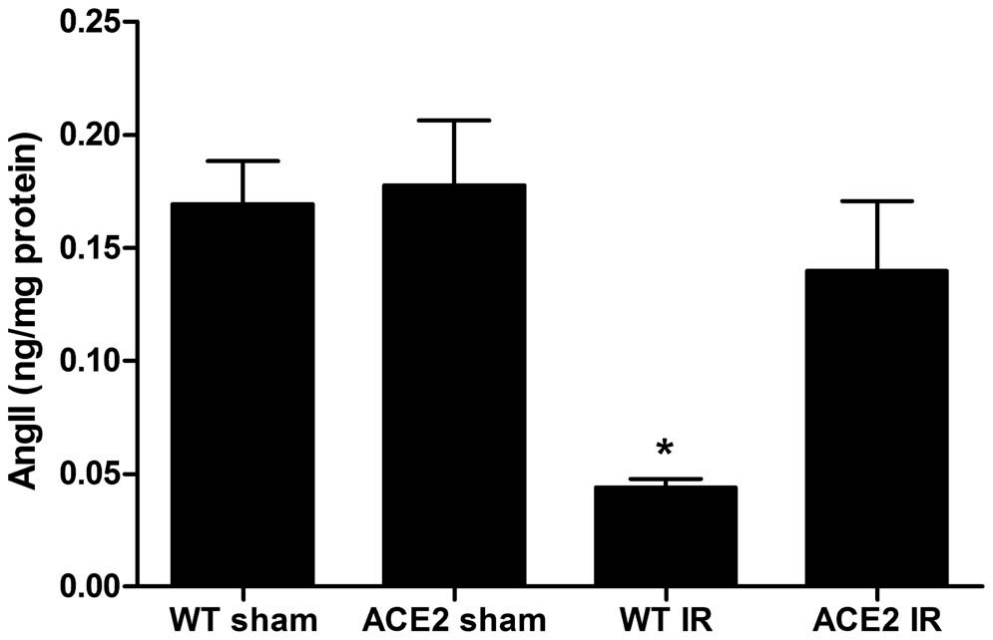
Levels of Ang II in kidney tissue in WT and ACE2 KO mice after sham or I/R surgery. Peptide levels were determined by ELISA and normalized to total protein. Results are presented as mean ± SE. n = 7 in WT sham and IR group; n = 8 in ACE2 sham and IR group. * p<0.05 vs. WT sham.

We then measured Ang II peptide levels in kidney tissues of the four groups of mice. Tissue Ang II levels were similar between sham WT and ACE2 KO mice, but after I/R, were significantly greater in the kidneys of ACE2 KO mice (n = 7) than WT mice (n = 8) (**Figure 13**).

### Evaluation of Renal Function Change After Bilateral I/R Injury

**Figure 14:**
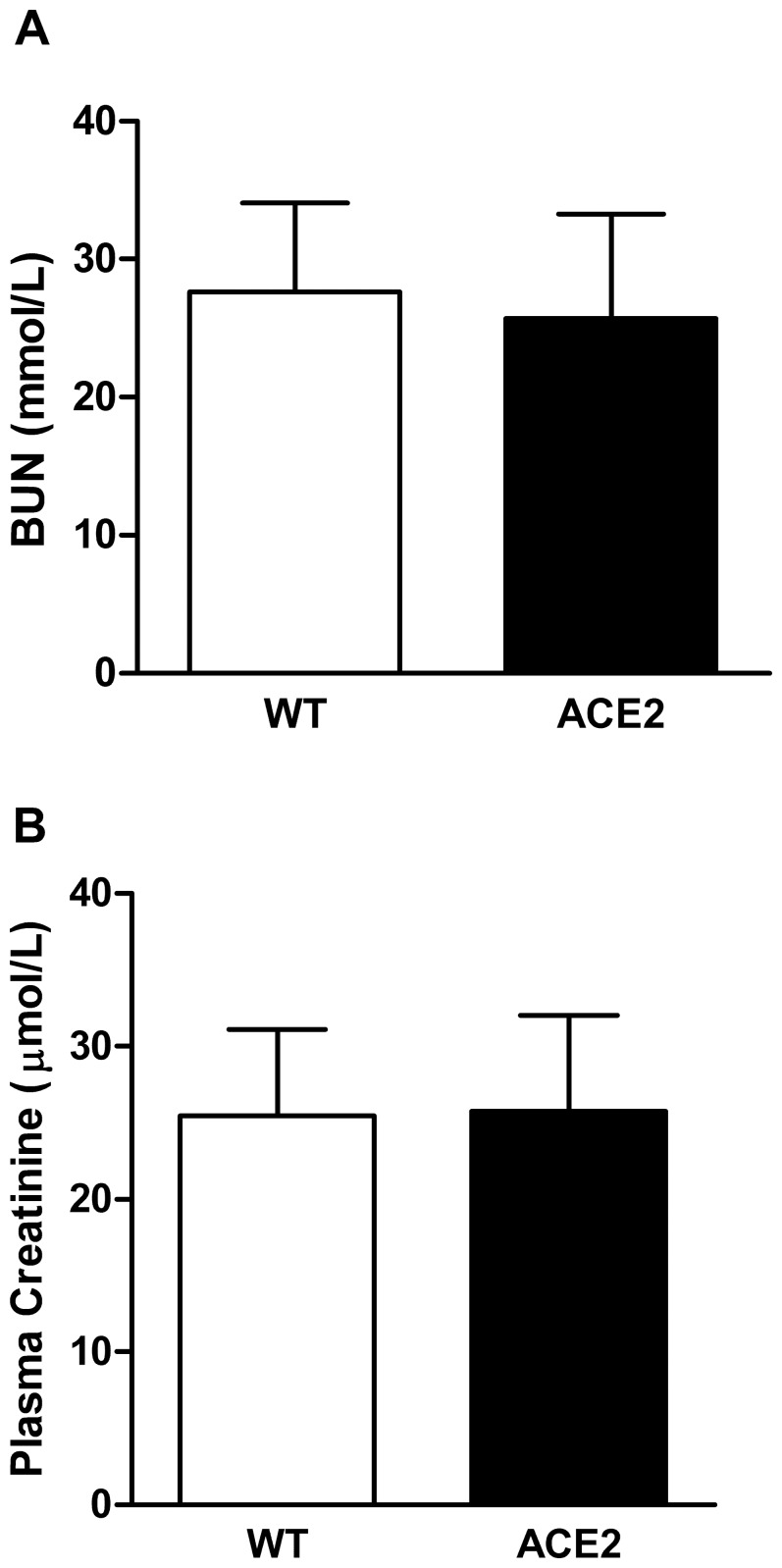
Plasma BUN and creatinine levels in WT and ACE2 KO mice following bilateral I/R. Plasma samples were obtained at sacrifice 48 hours after surgery. Frozen plasma samples were used for BUN and creatinine measurement. Results are presented as mean ± SE. n = 7 in WT group; n = 4 in ACE2 group.

To obtain a more robust measure of kidney function after I/R, we utilized a bilateral model of injury. There were no differences in plasma BUN and creatinine values between WT and ACE2 KO mice at 48 hours after reperfusion (**Figure 14**).

## Discussion

It has been reported that the intrarenal RAS is activated in the I/R model of AKI but the effect of loss of the gene for *ace2* on the kidney’s response to I/R has not been studied. We observed that deletion of the *ace2* gene significantly increases cellular inflammation, pro-inflammatory cytokine expression, apoptosis and oxidative stress following I/R. These data are the first to demonstrate a potentially protective effect of ACE2 on AKI.

I/R is a major cause of AKI with considerable morbidity and often leading to chronic kidney disease [27], [28]. The mechanisms of injury in AKI are complex and include ATP depletion with consequent cellular injury including necrosis or apoptosis [29], inflammatory cell recruitment and oxidative stress [4]–[6], [29]. The cortico-medullary junction including the S3 proximal tubule segment is especially vulnerable to ischemia because of intrinsic low oxygen tension coupled with elevated metabolic demand [4]. Besides tubular cell injury, diffuse endothelial cell damage has also been demonstrated in I/R, and both tubular and endothelial cells contribute to the recruitment of inflammatory cells [30]–[32]. Inflammation has become recognized as being a critical component of I/R injury. Various studies have shown important roles for neutrophils, T cells, B cells, macrophages and the complement pathway, although whether a dominant immune mechanism exists is not entirely clear [33]–[35]. Similarly, a variety of cytokines/chemokines secreted by injured kidney and infiltrating cells have been shown to be important to maintain the local inflammatory environment, while also causing direct cellular injury as with TNF-mediated apoptosis [36].

Most research in AKI-I/R has focused on these effectors of injury, namely, inflammatory cells, cytokines/chemokines, apoptosis or oxidative stress [37]–[39], but much less is known about potential upstream events including the formation of Ang II. The actions of Ang II overlap with mechanisms of I/R injury, namely inflammation and oxidative stress [40]. Moreover, because Ang II is elevated as early as 4 hours after I/R, mediators of I/R injury may at least in part be up-regulated by Ang II. While renin is known to be rate limiting for Ang II formation, the degradation to Ang-(1–7) by ACE2 is also a determinant of tissue Ang II concentrations. In this regard, a salutary effect of ACE2 has been shown in several models of CKD, including diabetic nephropathy, renal ablation, and most recently, unilateral ureteral obstruction, where a role for Ang II in potentiating injury is well established [20]–[23], [41]. A few early studies examining I/R have shown benefit by blocking Ang II, supporting the hypothesis that Ang II mediates at least some of kidney’s responses in this form of injury [42], [43]. Thus ACE2 could similarly affect the outcome of AKI.

Forty-eight hours after I/R, we found that the kidneys from ACE2 KO mice showed greater numbers of neutrophils, macrophages and T cells compared to the kidneys of WT mice. Although histologic injury scores tended to be higher in ACE2 KO mice, the differences were not statistically significant. Besides the dominant injury at the cortico-medullary junction area which included tubular necrosis, we also saw some injury in the cortex and medulla. Increased inflammatory cell infiltration was accompanied by higher levels of major pro-inflammatory cytokines and chemokines in the kidneys of ACE2 KO mice. IL-1β, IL-6, TNFα, MIP-2 and MCP-1 play important roles in immune function, including cell recruitment, maturation and activation [44], [45]. Resident cells are likely the main source of these cytokines/chemokines early after injury, whereas infiltrating cells may have a significant role at later time-points [46]. In addition, we found both apoptosis and oxidative stress, two processes that are associated with I/R and influenced by Ang II, to be exacerbated by the loss of *ace2* gene [37], [47]. The increase in oxidative stress may be a unifying aspect of the greater injury seen in the ACE2 KO mice, since hypoxia, infiltrating neutrophils and macrophages, and Ang II can each result in the generation of reactive oxygen species. Despite the greater inflammation and oxidative stress in the ACE2 KO mice, kidney function after 48 hours of reperfusion were similar in ACE2 KO and WT mice.

In evaluating the expression of RAS components, we found no differences in mRNA expression levels at baseline between WT and ACE2 KO mice. After I/R, there was a uniform trend towards increased mRNA expression levels in WT mice, but little to no change in ACE2 KO mice. In previous studies of I/R, Ang II levels were consistently elevated between 4 and 24 hours after reperfusion, and returned to baseline levels by 72 hours [11]–[13]. We confirmed increased Ang II levels in ACE KO compared to WT mice at 48 hours after I/R, although by this time, Ang II levels had also decreased compared to baseline. The decrease in Ang II levels is consistent with decreased ACE staining that was seen at this time-point.

We did not explore the mechanism(s) responsible for the observed effect of loss of ACE2 on I/R, which is a limitation of our study. It is tempting to speculate that increased Ang II concentrations played a role, especially since Ang II levels were elevated compared to WT mice even at 48 hours after I/R. As regards Ang (1–7), it is notable that a recent study showed that administration of Ang-(1–7) worsened kidney I/R injury *in vivo* and activated NF-κB in cultured tubular cells *in vitro* [48], [49]. On the other hand, there is also evidence that Ang-(1–7) counters the effect of Ang II in cultured tubular cells and mesangial cells [50], [51]. Another mechanism might include des-Arg bradykinin, because absent ACE2 would potentially cause an increase in this active bradykinin metabolite [52]. Additional experiments will be required to better define the mechanism responsible for the protective effect of ACE2 in I/R.

Another limitation of our study is the lack of detailed assessment of recovery from I/R injury between the two groups of mice. We surmised that increased inflammation and oxidative stress seen in the ACE2 KO mice might translate into less complete recovery compared to the WT mice. We used Ki-67 as an indicator of tissue recovery and found the degree of cellular proliferation to be similar between the ACE2 KO and WT mice at 48 hours after I/R.

In conclusion, our data show that ACE2 is a determinant of the renal response to I/R. Pathologic examination of kidneys showed increased inflammation, apoptosis and oxidative stress in ACE2 KO mice compared to WT mice. Administration of recombinant human ACE2 has been shown to mitigate injury in various disease models of elevated Ang II, and may also hold promise in the treatment of I/R-induced acute kidney injury [53], [54].
